# Locoregional treatment of breast cancer during pregnancy

**DOI:** 10.1007/s10397-014-0860-6

**Published:** 2014-09-30

**Authors:** Antonio Toesca, Oreste Gentilini, Fedro Peccatori, Hatem A. Azim, Frederic Amant

**Affiliations:** 1 Division of Breast Surgery, European Institute of Oncology, Via Ripamonti 435, 20141 Milan, Italy; 2 Department of Gynecological Oncology, European Institute of Oncology, Milan, Italy; 3 Department of Medicine, BrEAST Data Centre, Institut Jules Bordet, Université Libre de Bruxelles, 1000 Brussels, Belgium; 4 Multidisciplinary Breast Center, Leuven Cancer Institute (LKI), UZ Gasthuisberg, Katholieke Universiteit Leuven, Leuven, Belgium

**Keywords:** Breast cancer, Pregnancy, Chemotherapy, Radiation therapy

## Abstract

The management of patients with breast cancer during pregnancy is very demanding and it should be better performed in highly qualified and experienced centers. Referral to institutes and physicians trained in this special clinical scenario allows reducing the risk of both overtreating and undertreating the patients. Moreover, patients can receive appropriate information regarding safety of treatments without old-fashioned *taboo*. The purpose of the current paper is to discuss the main issues concerning surgical management and in general locoregional treatment of patients diagnosed with breast cancer and treated during gestation, focusing on those women who chose to continue their pregnancy. We cover the issues regarding type of breast surgery, radiation therapy, immediate reconstruction during mastectomy, and management of the axilla.

## Introduction and general considerations

Breast cancer diagnosed during pregnancy (BCdP), defined as breast cancer which develops either during or within 1 year after pregnancy, is expected to become even more common, since women often delay childbearing to their thirties and forties when breast cancer rates tend to increase. Some studies have found that BCdP is more commonly diagnosed at an advanced stage because of increased breast density, making clinical examinations and mammography more difficult to interpret [1–3].

Prognosis is influenced by treatment options, either local or systemic, which might be limited by the concern of harming the fetus and conditioned by gestational age. Therefore, it is important to clarify that most part of therapies can be safely administered to pregnant patients as well giving the opportunity to the mother to receive optimal treatments [4–6]. Azim et al. [1] reported in their metaanalysis of pregnancy-associated breast cancer that there was a poorer breast cancer outcome for women diagnosed in the postpartum period compared with those diagnosed with breast cancer during pregnancy. This is not in contrast with Amant et al. [7] who reported on the prognosis of women with primary breast cancer diagnosed during pregnancy and note similar overall survival compared with general population of non-pregnant patients. Many studies in the past have considered the two groups (breast cancer during pregnancy and post-partum breast cancer) as part of the same condition, and this could be the reason for the controversial results on prognosis.

Though, the occurrence of BCdP represents a dramatic condition for the patient, her family, and sometimes her physician mostly if the lattest is not carefully and specifically trained on this special clinical scenario. In fact, the management of BCdP requires a collaborative team effort to provide the best medical options and most effective psychosocial support.

The purpose of the current paper is to discuss the main issues concerning surgical management and in general loco-regional treatment of patients diagnosed with breast cancer during pregnancy (BCdP). As treatment of breast cancer during lactation does not imply special major problems in terms of availability of treatments, our paper will focus only on patients with breast cancer diagnosed and treated during gestation. Moreover, this special clinical scenario is still infrequent, and therefore, some of the recommendations may necessarily have only a low level of evidence (expert opinion). Before treatment, it is important to discuss and inform the woman and her family about the maternal prognosis and treatment options as well as the potential impact on pregnancy and delivery, according to different staging. As interruption of pregnancy give access to therapies as in a non pregnant woman, the multidisciplinary team have to discuss differences with the patients in case of continuation of the pregnancy.

Our considerations will be restricted to those women who choose to continue their pregnancy, and all the issues related to voluntary interruption of pregnancy will not be included. As a general statement, the patients should be made aware that interruption of pregnancy by itself does not seem to improve outcome of patients [8].

## Breast conserving surgery and the problem of delaying radiation therapy

Historically, mastectomy was considered the standard surgical procedure in pregnant patients with breast cancer [9].

Actually, due to a frequent diagnostic delay, patients with BCdP often present with large tumors requiring radical surgery. Modern studies report a mean diagnostic delay during pregnancy and lactation ranging from 1 to 3 months with a median tumor size at diagnosis of 3.5 cm [9]. Nevertheless, in our opinion, it is important to inform the patient that mastectomy is not mandatory for the treatment of breast cancer just because of the presence of pregnancy by itself [10, 11].

The published experience on breast conservation is so far limited, but all the available data seem to go in the same direction supporting safety and feasibility of breast conservation with good prognostic results in terms of local control [12–14]. In the experience of European Institute of Oncology of Milan [12], tumor size and rate of axillary metastases in patients with BCdP were lower than in previous reports [13], probably because of the increased awareness among both patients and physicians. This earlier stage of presentation (median tumor size 2.4 cm) enabled a higher rate of breast-conserving procedures (15 of 21 patients) even though it has to be pointed out that all the six patients who were diagnosed during the first trimester opted for termination of pregnancy. After a short-term median follow-up (24 months), there were no intra-breast tumor recurrences. Kuerer et al. reported similar survival rates between patients treated with breast-conserving surgery and those treated with mastectomy [14].

As a general recommendation, breast conservation can be safely performed, whenever possible, in women diagnosed during the third trimester, as radiotherapy can be postponed until after delivery without major concerns about a possible detrimental delay.

The concurrent diagnosis of breast cancer and an unexpected early pregnancy represents the most challenging treatment scenario. It is considered that abortion is not a therapeutic procedure in these cases [13], but termination of pregnancy can be considered in order to facilitate completion of treatment. For patients at the first trimester who desire to continue the pregnancy, treatment is possible but there is a limited number of options during the first weeks of gestation. In fact, chemotherapy is prohibited during the first trimester, and endocrine treatments are not feasible [13, 15]. Surgery is safe at any time and during the first trimester as well [16], but breast conservation performed during a very early gestational age is associated with a long delay in postoperative radiotherapy. Unfortunately, there is limited and retrospective experience published on the delayed radiotherapy after breast conservation and its effect on outcome. In a study evaluating 568 patients with T1–T2 N0 breast cancer who underwent lumpectomy and radiotherapy without systemic treatment, similar rate of recurrence was reported in node negative patients when radiotherapy starts up to 16 weeks after definitive surgery after a median follow-up of 11.2 years [17]. Another retrospective study reported on 13,907 patients aged 65 years or older with stage I–II breast cancer who underwent lumpectomy and radiotherapy taken from the Surveillance, Epidemiology, and End Results (SEER)–Medicare database. The authors concluded that delays of >3 months were associated with poor survival, even though older age, black race, advanced stage, more comorbidities, and being unmarried were associated with longer time intervals between surgery and RT, and therefore, it is not clear whether the association is causal or due to confounding factors [18]. Chen et al. [19] performed a systematic review on the relationship between waiting time for radiotherapy and clinical outcomes with special attention on local recurrence. In this meta-analysis considering 20 high quality studies that had adequately controlled for confounding factors, a significant increase of local failure was demonstrated with increasing waiting times. The authors subsequently converted the relative risks derived from the meta-analysis into estimates of the increment in risk attributable to 1 month of the waiting time for RT and this translated into an absolute increase in the risk of local recurrence of 1.0 % per month of delay of staring RT.

Trying to be practical despite these controversial data, it is very likely that pregnant patients with breast cancer will undergo adjuvant chemotherapy after surgery as this is the only adjuvant possible treatment during gestation. Actually, most part of nonpregnant patients undergoing adjuvant chemotherapy usually receive radiotherapy after more than 6 months, and in these patients, the delay of administration does not represent an issue of major concern.

Basically, we suggest that in patients at the second or third trimester, the surgical approach applied to women with BCdP should not significantly differ from the policy applied to nonpregnant women, and the delay in administering RT is probably similar to what happens to nonpregnant patients.

In a patient at the first trimester who wants to continue the pregnancy and also wishes to conserve the breast, all these issues have to be carefully discussed, and the patient has to be informed that a possible increased risk of local recurrence should be considered, even though this is difficult to quantify because of the lack of clear data. The patients should also be reassured that in nonpregnant patients receiving chemotherapy, radiotherapy is usually given 6 months after surgery.

## External beam radiation therapy

Embryonic exposure resulting from breast radiotherapy with a dose of 0.1 Gy in the first trimester, during organogenesis, increases the risk of malformations and can cause mental retardation [20, 21].

The dose to a fetus resulting from tangential breast irradiation, measured using anthropomorphic phantoms simulating the geometry of a pregnant woman, has been calculated for the first, second, and third trimester of gestation [20]. The dose increased as the pregnancy became more advanced, because of the increased proximity of the fetus to the primary irradiation field. With shielding a 50–75 % dose reduction can be achieved [22, 23]. These data are applicable for all the X-ray energies from 4 to 10 MV used for breast radiotherapy. Thus, during the first and the second trimester of pregnancy, the fetal irradiation dose seems to be lower than the threshold values associated organ malformations. During the third trimester, however, the dose seems to exceed this threshold. In addition, in utero irradiation at all gestational ages may increase the risk of cancer during childhood [20]. A conservative estimate of the lifetime risk of radiation induced by fetal exposure to 0.01 Gy is about one in 1700 cases [22].

Successful radiotherapy of breast cancer during pregnancy and birth of healthy children has been reported [24–28]. The short-term fetal outcome following radiotherapy for BCdP has been recently documented. After a median follow up of 37 months, Luis et al. calculated 13/109 adverse outcomes, including spontaneous abortions (*n* = 2), perinatal death (*n* = 5), stillbirth (*n* = 1), hypospadia (*n* = 1), learning problem and scoliosis (*n* = 1), sensory hearing loss (*n* = 1), attention deficit disorder with delayed coordination (*n* = 1), undescended left testicle, and an uncomplicated ventricular septal defect (*n* = 1) [27]. Where available (*n* = 4), the estimated fetal dose was below the threshold dose (<0.1 Gy). Of the 24 patients treated for breast cancer, 3 had an adverse fetal outcome: 2 perinatal deaths were described after chest wall/axilla irradiation and one spontaneous abortion after lumbar spine irradiation (30 Gy) at 10 weeks of gestation for metastatic disease [29]. Overall, the fetal outcome is poorly documented and it is difficult to define the role of radiotherapy when an adverse outcome is noted.

Therefore, radiotherapy is considered relatively safe only during the first and second trimester of pregnancy but based on theoretical assumptions and few experiences. Better clinical data are needed and every single case should be discussed with a patient and by a multidisciplinary team, tailoring as much as possible every single case [11].

## Is partial breast irradiation possible during pregnancy?

The strength and the attractiveness of accelerated partial breast irradiation (APBI) techniques for breast cancer are reducing the volume treated, with potential decrease of normal tissue toxicity, and reducing the treatment time [30]. In response to the increasing use of APBI off clinical trial several consensus statements from different panels have been published regarding the appropriate use of partial breast irradiation in nonpregnant breast cancer patients. The National Comprehensive Cancer Network (NCCN) guidelines published in 2011 [31] open the possibility to patients to be given APBI according to criteria identified by American Society for Radiation Oncology (ASTRO) consensus for the “suitable” group which includes only women aged >60 years. Therefore, the application of APBI remains controversial in young patients with breast cancer due to the increased local recurrence rate after breast conservation in this subset of patients. Nevertheless, the issues concerning on one hand safety and on the other hand the possible risks of delaying radiotherapy in pregnant patients with breast cancer make PBI theoretically attractive as an alternative option.

Electron beam intraoperative radiotherapy (ELIOT) is a new technique permitting breast radiotherapy to be completed in a single session. Since ELIOT is associated with much reduced irradiation to non-target tissues, Galimberti el at. carried out a study on nonpregnant breast cancer patients to estimate doses to the uterus during ELIOT [32].

The authors performed in vivo dosimetry with thermoluminescence radiation detectors (TLDs) in 15 premenopausal patients receiving ELIOT to the breast (prescribed dose 21 Gy) using two mobile linear accelerators. The TLDs were positioned subdiaphragmatically on the irradiated side, at the medial pubic position, and within the uterus. A shielding apron (2-mm lead equivalent) was placed over the viscera from the subcostal to the subpubic region. TLDs showed mean doses of 0.37 Gy (range 0.01–0.85 Gy) at subdiaphragm, 0.09 Gy (range 0.003–0.02 Gy) pubic, and 0.17 Gy (range 0.06–0.32 Gy) in utero, for beam energies in the range 5–9 MeV. These findings indicate that ELIOT with a mobile linear accelerator and shielding apron would be safe for the fetus, as doses of a few Gy are not associated with measurable increased risk of fetal damage, and the threshold dose for deterministic effects is estimated at 0.1–0.2 Gy.

Intraoperative radiotherapy could reduce fetal dose, and for this result so attractive for pregnant management, there are limitations and doubts about the efficacy of PBI in young patients with breast cancer. Nevertheless, we believe that this might be a further option to offer to pregnant patients with a small breast cancer diagnosed at a very early gestational age and who are motivated to continue the pregnancy after a thorough explanation of a possible increase in local recurrence if compared to WBRT. Always an estimation of the fetal exposure should be assessed by a physicist in order to assess fetal safety.

## Mastectomy and immediate breast reconstruction

In the recent past, mastectomy has been considered the treatment of choice for pregnant patients with breast cancer [9]. To date, despite breast conservation can be considered, a considerable proportion of patients to date still require a mastectomy due to the large tumor size at presentation.

In 2010, a European Consensus on the management of breast cancer during pregnancy discouraged immediate breast reconstruction during pregnancy due to lack of data and recommended prosthetic implant-based reconstruction after delivery [11]. In fact, at the moment, there are no available data concerning immediate breast reconstruction (IBR) in pregnant patients undergoing mastectomy for breast cancer. Nevertheless, it is well known that IBR decreases the psychological impact of mutilation, provides superior esthetic outcome and better patient and physician satisfaction compared to delayed reconstruction [33–35].

Therefore, the obvious advantages of immediate breast reconstruction lead us to explore the possibility to consider an IBR whenever possible even in pregnant patients, and in our view, pregnant breast cancer patients should not be denied by definition the opportunity to undergo immediate breast reconstruction after mastectomy. At the European Institute of Oncology, we usually suggest a tissue expander which is a straightforward technique not significantly increasing operating time and risk of complications. Lohsiriwat et al. [36] reported the first analysis of 78 patients who underwent immediate breast reconstruction with expander following mastectomy for breast cancer diagnosed during pregnancy describing an excellent pregnancy outcomes without obstetrical complications after surgery. Moreover, the unpredictable physiologic changes of the breast during and after pregnancy, makes not suitable IBR with definitive implant and contralateral reshaping. IBR by autologous tissue should not be considered for the long operative time and increased risk of blood loss and postoperative complications.

## Sentinel lymph node biopsy

After initial concern for a safety issue, it is now widely agreed that sentinel lymph node biopsy (SLNB) for staging of the regional lymph nodes can be performed safely during pregnancy [37, 38].

In 2000, Nicklas and Baker [39] suggested that SLNB can be safely performed in pregnancy since the entire radioisotope injected (13.5 to16 MBq of double-filtered 99mTc sulfur colloid) remains trapped at the injection site on the breast or within the lymphatics. Morita et al. [40] stated that receiving a whole-body dose from activity 13.5 to 16 MBq in the breast, the dose of radiation exposure to the unborn child would be exceedingly low. Some authors [39, 41, 42] reported that the estimated absorbed dose to the fetus/embryo per unit activity of 99mTc-HSA administered intravenously to the mother is 5.1 mGy/MBq. Dosimetric evaluations reported in the literature as well as data from a simulation study gave evidence of negligible risks to the fetus [43]. Gentilini et al. performed a simulation in vivo study in order to investigate safety of lymphoscintigraphy in terms of radiation risk and estimate of the possible absorbed doses to the fetus with a single peritumoral injection of 99mTc-labeled human albumin colloid particles (99mTc-HSA nanocolloids) in a volume of 0.2 ml 16–18 h before the surgical intervention. The injected activity was found to be concentrated only in the injection site and in the lymph nodes, demonstrating negligible irradiation to other tissues, organs, and the absence of radiotracer uptake in the pelvis after 15 min. In 23 of 26 nonpregnant patients studied, all absorbed dose measurements were lower than the sensitivity of the thermoluminescent dosimeters used (<10 mGy); in the remaining three patients, the absorbed doses at the level of epigastrium, umbilicus, and hypogastrium ranged from 0.03 to 0.32 Gy. The total activity excreted in the urine within the first 16 h (time between injection and operation) was <2 % of the injected activity. The biological pharmacokinetic data showed that a very small amount of the injected activity is circulating in the blood pool and excreted by the urinary system confirming that the level of radioactivity in the body is absolutely negligible at each time point studied after the administration, proving that there is a negligible risk to the fetus [37]. This level is far less than the National Council on Radiation Protection and Measurements limit to pregnant women [44].

Experiences derived from treatment of melanoma or breast cancer who underwent lymphatic mapping during pregnancy have not shown birth defects or discernible malformations in born [38].

Gentilini et al. reported data from 12 pregnant patients with breast cancer who underwent lymphoscintigraphy and SLNB, focusing on the outcomes of the pregnancies. Eleven babies were born with normal weight and no malformations and after a median follow up of 32 months (6–83 months), were doing well. One baby had a diagnosis of ventricular septal defect (VSD) and was operated on at the age of 3 months because of the onset of cardiac failure. However, VSD was demonstrated with ultrasound before the lymphoscintigraphy procedure and therefore cannot be attributed to the injection of the radioactive tracer.

As a practical recommendation, it is advisable to inject colloid in the morning (1-day protocol) in order to reduce time and dose of radiation exposure.

Blue dye should not be used during pregnancy as its use has a possible risk of an allergic or anaphylactic maternal reaction, which can be harmful for the fetus [43]. Isosulfan blue has a possible risk (1 %) of an allergic and anaphylactic reaction, which can increase the risk of harm to the fetus. Methylene blue is contraindicated in the pregnant patients during first trimester because of known teratogenic effect of jejunal atresia due to of vasoconstrictive effects in blocking nitric oxide [43].

## Concluding remarks

Surgery can be safely performed during pregnancy and during the first trimester as well. Mastectomy should not be recommended just because of the pregnancy itself, and breast conservation should be discussed whenever possible. In patients operated during the third or even the second trimester, radiation therapy can be safely postponed after delivery [11]. The risk of a possible too long delay of radiation therapy in case of surgery performed at a very early gestational age should be taken into account and all the options should be considered according to patient’s preference. However, virtually, all patients need adjuvant chemotherapy, bridging the gap between surgery and radiotherapy. Partial breast irradiation, especially with electrons (ELIOT) might be an interesting option in the future even if at the moment there is lack of data and some doubts might be raised regarding treatment of young patients in terms of increased risk of local recurrence. For those patients requiring mastectomy, an immediate breast reconstruction with tissue expander can be performed as it does not excessively increase operative time and risk of complications. Lymphoscintigraphy and sentinel node biopsy by the use of 99mTc is safe in pregnant patients as well.

## References

[1] Azim HA, Santoro L, Russell-Edu W, Pentheroudakis G, Pavlidis N, Peccatori FA (2012). Prognosis of pregnancy-associated breast cancer: a meta-analysis of 30 studies. Cancer Treat Rev.

[2] Ishida T, Yokoe T, Kasumi F (1992). Clinicopathologic characteristics and prognosis of breast cancer patients associated with pregnancy and lactation: analysis of case–control study in Japan. Jpn J Cancer Res.

[3] Anderson BO, Petrek JA, Byrd DR, Senie RT, Borgen PI (1996). Pregnancy influences breast cancer stage at diagnosis in women 30 years of age and younger. Ann Surg Oncol.

[4] Azim HA, Botteri E, Renne G (2012). The biological features and prognosis of breast cancer diagnosed during pregnancy: a case–control study. Acta Oncol.

[5] Amant F, Loibl S, Neven P, Van Calsteren K (2012). Breast cancer in pregnancy. Lancet.

[6] Amant F, Van Calsteren K, Halaska MJ, Gziri MM, Hui W, Lagae L, Willemsen MA, Kapusta L, Van Calster B, Wouters H, Heyns L, Han SN, Tomek V, Mertens L, Ottevanger PB (2012). Long-term cognitive and cardiac outcomes after prenatal exposure to chemotherapy in children aged 18 months or older: an observational study. Lancet Oncol.

[7] Amant F, von Minckwitz G, Han SN, Bontenbal M, Ring AE, Giermek J, Wildiers H, Fehm T, Linn SC, Schlehe B, Neven P, Westenend PJ, Müller V, Van Calsteren K, Rack B, Nekljudova V, Harbeck N, Untch M, Witteveen PO, Schwedler K, Thomssen C, Van Calster B, Loibl S (2013). Prognosis of women with primary breast cancer diagnosed during pregnancy: results from an international collaborative study. J Clin Oncol.

[8] Loibl S, Han SN, von Minckwitz G, Bontenbal M, Ring A, Giermek J, Fehm T, Van Calsteren K, Linn SC, Schlehe B, Gziri MM, Westenend PJ, Müller V, Heyns L, Rack B, Van Calster B, Harbeck N, Lenhard M, Halaska MJ, Kaufmann M, Nekljudova V, Amant F (2012). Treatment of breast cancer during pregnancy: an observational study. Lancet Oncol.

[9] Woo JC, Yu T, Hurd TC (2003). Breast cancer in pregnancy: a literature review. Arch Surg.

[10] Schwartz GF, Veronesi U, Clough K, Dixon JM, Fentiman IS, Heywang-Köbrunner SH, Holland R, Hughes KS, Margolese R, Olivotto IA, Palazzo JP, Solin LJ (2006) Proceedings of the Consensus Conference on Breast Conservation, April 28 to May 1, 2005, Milan, Italy Cancer; 107(2): 242–50 10.1002/cncr.21988 16770785

[11] Amant F, Deckers S, Van Calsteren K, Loibl S, Halaska M, Brepoels L, Beijnen J, Cardoso F, Gentilini O, Lagae L, Mir O, Neven P, Ottevanger N, Pans S, Peccatori F, Rouzier R, Senn HJ, Struikmans H, Christiaens MR, Cameron D, Du Bois A (2010). Breast cancer in pregnancy: recommendations of an international consensus meeting. Eur J Cancer.

[12] Gentilini O, Masullo M, Rotmensz N (2005). Breast cancer diagnosed during pregnancy and lactation: biological features and treatment options. Eur J Surg Oncol.

[13] Berry DL, Theriault RL, Holmes FA (1999). Management of breast cancer during pregnancy using a standardized protocol. J Clin Oncol.

[14] Kuerer H, Gwyn K, Ames F (2002). Conservative surgery and chemotherapy for breast carcinoma during pregnancy. Surgery.

[15] Cardonick E, Iacubucci A (2004). Use of chemotherapy during pregnancy. Lancet Oncol.

[16] Duncan PG, Pope WDB, Cohen MM, Greer N (1986). Fetal risk of anesthesia and surgery during pregnancy. Anesthesiology.

[17] Vujovic O, Cherian A, Dar AR, Stitt L, Perera F (2006). Eleven-year follow up results in the delay of breast irradiation after conservative surgery in node-negative breast cancer patients. Int J Radiat Oncol Biol Phys.

[18] Hershman DL, Wang X, McBride R, Jacobson JS, Grann VR, Neugut AI (2006). Delay in initiating adjuvant radiotheraphy following breast conservation and its impact on survival. Int J Radiat Oncol Biol Phys.

[19] Chen Z, King W, Pearcey R, Kerba M, Mackillop WJ (2008). The relationship between waiting time for radiotherapy and outcome: a systematic review of the literature. Radiother Oncol.

[20] Mazonakis M, Varveris H, Damilakis J (2003). Radiation dose to conceptus resulting from tangential breast irradiation. Int J Radiat Oncol Biol Phys.

[21] International Commission on Radiological Protection (1991). Recommendations of the International Commission on Radiological Protection, ICRP Publication 60.

[22] Kal HB, Struikmans H (2005). Radiotherapy during pregnancy: fact and fiction. Lancet Oncol.

[23] Han B, Bednarz B, Xu XG (2009). A study of the shielding used to reduce leakage and scattered radiation to the fetus in a pregnant patient treated with a 6-MV external X-ray beam. Health Phys.

[24] Van der Giessen PH (1997). Measurement of the peripheral dose for the tangential breast treatment technique with Co-60 gamma radiation and high energy X-rays. Radiother Oncol.

[25] Ngu SL, Duval P, Collins C (1992). Foetal radiation dose in radiotherapy for breast cancer. Australas Radiol.

[26] Antypas C, Sandilos P, Kouvaris J (1998). Fetal dose evaluation during breast cancer radiotherapy. Int J Radiat Oncol Biol Phys.

[27] Luis SA, Christie DR, Kaminski A (2009). Pregnancy and radiotherapy: management options for minimising risk, case series and comprehensive literature review. J Med Imaging Radiat Oncol.

[28] Kouvaris JR, Antypas CE, Sandilos PH, Plataniotis GA, Tympanides CN, Vlahos LJ (2000). Postoperative tailored radiotherapy for locally advanced breast carcinoma during pregnancy: a therapeutic dilemma. Am J Obstet Gynecol.

[29] Mulvihill JJ, McKeen EA, Rosner F, Zarrabi MH (1987). Pregnancy outcome in cancer patients. Experience in a large cooperative group. Cancer.

[30] Orecchia R, Leonardo MC (2011). Intraoperative radiation therapy: is it a standard now?. Breast.

[31] NCC guidelines. http://www.nccn.org/professionals/physician_gls/PDF/breast.pdf

[32] Galimberti V, Ciocca M, Leonardi MC (2009). Is electron beam intraoperative radiotherapy (ELIOT) safe in pregnant women with early breast cancer? In vivo dosimetry to assess fetal dose. Ann Surg Oncol.

[33] Morrow M (2009). Surgeon recommendations and receipt of mastectomy for treatment of breast cancer. JAMA.

[34] Al-Ghazal SK, Sully L, Fallowfield L, Blamey RW (2000). The psychological impact of immediate rather than delayed breast reconstruction. Eur J Surg Oncol.

[35] Fernandez-Delgado J, Lopez-Pedraza MJ, Blasco JA (2008). Satisfaction with and psychological impact of immediate and deferred breast reconstruction. Ann Oncol.

[36] Lohsiriwat V, Peccatori FA, Martella S, Azim HA, Sarno MA, Galimberti V, De Lorenzi F, Intra M, Sangalli C, Rotmensz N, Pruneri G, Renne G, Schorr MC, Nevola Teixeira LF, Rietjens M, Giroda M, Gentilini O (2013). Immediate breast reconstruction with expander in pregnant breast cancer patients. Breast.

[37] Gentilini O, Cremonesi M, Trifiro G (2004). Safety of sentinel node biopsy in pregnant patients with breast cancer. Ann Oncol.

[38] Gentilini O, Cremonesi M, Toesca A (2010). Sentinel lymph node biopsy in pregnant patients with breast cancer. Eur J Nucl Med Mol Imaging.

[39] Du Bois A, Meerpohl HG, Gerner K (1993). Effect of pregnancy on the incidence and course of malignant diseases. Geburtshilfe Frauenheilkd.

[40] Morita ET, Chang J, Leong SP (2000). Principles and controversies in lymphoscintigraphy with emphasis on breast cancer. Surg Clin N Am.

[41] Russell JR, Stabin MG, Sparks RB, Watson E (1997). Radiation absorbed dose to the embryo/fetus from radiopharmaceuticals. Health Phys.

[42] Russell JR, Stabin MG, Sparks RB (1997). Placental transfer of radiopharmaceuticals and dosimetry in pregnancy. Health Phys.

[43] Khera SY, Kiluk JV, Hasson DM (2008). Pregnancy-associated breast cancer patients can safely undergo lymphatic mapping. Breast J.

[44] Pandit-Taskar N, Dauer LT, Montgomery L, Germain SJ, Zanzonico PB, Divgi CR (2006). Organ and fetal absorbed dose estimates from 99 mTc-sulfur colloid lymphoscintigraphy and sentinel node localization in breast cancer patients. J Nucl Med.

